# Studying the Structure and Properties of Epoxy Composites Modified by Original and Functionalized with Hexamethylenediamine by Electrochemically Synthesized Graphene Oxide

**DOI:** 10.3390/nano14070602

**Published:** 2024-03-28

**Authors:** Anton Mostovoy, Amirbek Bekeshev, Sergey Brudnik, Andrey Yakovlev, Andrey Shcherbakov, Nurgul Zhanturina, Arai Zhumabekova, Elena Yakovleva, Vitaly Tseluikin, Marina Lopukhova

**Affiliations:** 1 Laboratory of Modern Methods of Research of Functional Materials and Systems, Yuri Gagarin State Technical University of Saratov, Polytechnichskaya Str., 77, 410054 Saratov, Russia; 2 Laboratory of Polymer Composites, K. Zhubanov Aktobe Regional State University, Aliya Moldagulova Avenue 34, Aktobe 030000, Kazakhstan; amirbek2401@gmail.com; 3 Department of Chemistry and Chemical Technology of Materials, Yuri Gagarin State Technical University of Saratov, Polytechnichskaya Str., 77, 410054 Saratov, Russia; sbrudraboch@gmail.com (S.B.); aw_71@mail.ru (A.Y.); 4 Laboratory of Support and Maintenance of the Educational Process, Yuri Gagarin State Technical University of Saratov, Polytechnichskaya Str., 77, 410054 Saratov, Russia; 5 Department of Physics, K. Zhubanov Aktobe Regional State University, Aliya Moldagulova Avenue 34, Aktobe 030000, Kazakhstan; nzhanturina@mail.ru; 6 Department of Chemistry, Chemical Technology and Ecology, Kazakh University of Technology and Business, Kayym Mukhamedkhanov Str., Building 37 A, Astana 010000, Kazakhstan; zhumabekova_ak@mail.ru; 7 Department of Ecology and Technosphere Safety, Yuri Gagarin State Technical University of Saratov, Polytechnichskaya Str., 77, 410054 Saratov, Russia; elenayakovleva@list.ru; 8 Department of Technology and Equipment for Chemical, Oil and Gas and Food Industries, Yuri Gagarin State Technical University of Saratov, Polytechnichskaya Str., 77, 410054 Saratov, Russia; tseluikin@mail.ru; 9 Department of Economics and Humanitarian Sciences, Yuri Gagarin State Technical University of Saratov, Polytechnichskaya Str., 77, 410054 Saratov, Russia; mlopuhova@yandex.ru

**Keywords:** graphene oxide, electrochemical synthesis, hexamethylenediamine, epoxy resin, modification, structure, properties

## Abstract

In this study, we used multilayer graphene oxide (GO) obtained by anodic oxidation of graphite powder in 83% sulfuric acid. The modification of GO was carried out by its interaction with hexamethylenediamine (HMDA) according to the mechanism of nucleophilic substitution between the amino group of HMDA (HMDA) and the epoxy groups of GO, accompanied by partial reduction of multilayer GO and an increase in the deformation of the carbon layers. The structure and properties of modified HMDA-GO were characterized using research methods such as scanning electron microscopy (SEM), Fourier transform infrared spectroscopy (FTIR), X-ray diffraction spectroscopy and Raman spectroscopy. The conducted studies show the effectiveness of using HMDA-OG for modifying epoxy composites. Functionalizing treatment of GO particles helps reduce the free surface energy at the polymer–nanofiller interface and increase adhesion, which leads to the improvement in physical and mechanical characteristics of the composite material. The results demonstrate an increase in the strength and elastic modulus in bending by 48% and 102%, respectively, an increase in the impact strength by 122%, and an increase in the strength and elastic modulus in tension by 82% and 47%, respectively, as compared to the pristine epoxy composite which did not contain GO-HMDA. It has been found that the addition of GO-HMDA into the epoxy composition initiates the polymerization process due to the participation of reactive amino groups in the polymerization reaction, and also provides an increase in the thermal stability of epoxy nanocomposites.

## 1. Introduction

Since the discovery of graphene [1], the relevance of the development of the scientific component of this allotropic modification of carbon has been gaining importance. In particular, the development of the chemistry of graphene derivatives, graphene oxide (GO) being one of such graphene forms, has received great interest [2]. One of the ways to change the functional properties of GO is covalent or non-covalent modification of GO [3,4,5], which is essential for the creation of various composite materials [6,7]. Interest in the derivatization of GO stems from the wide possibilities of regulating the chemical and physical properties of the material which are due to the addition of a certain amount of both organic functional groups on the basal plane and on the edge sections of GO and of inorganic compounds [8,9,10,11]. There has been growing interest in the modification of organic structures containing an amino group, especially aliphatic and/or aromatic amines, to change the structure of the GO surface [12,13,14].

Thus, in [15], synthesis by a two-stage liquid-phase treatment with bromic acid and ammonia solution is described. Using X-ray photoelectron spectroscopy (XPS), the degree of amination of the resulting aminated graphene oxide is defined, which was about 4 atomic %, the C/O ratio reached 8.8. The authors describe the chemical reactivity of the added amines, which is confirmed by the successful testing of the covalent modification of the resulting aminated graphene with 3-chlorobenzoyl chloride.

Chakraborty et al. [16] have demonstrated the possibility of amination of GO by a one-step synthesis method using short-chain alkylamine n-butylamine without applying toxic precursors such as thionyl/acyl chlorides. The method also eliminates the stage of reducing other GO functional groups. The presence of nitrogen on the surface of graphene oxide was confirmed using energy dispersive analysis and X-ray photoelectron spectroscopy. The XPS spectrum of N1s further confirmed the presence of nitrogen in the form of a secondary amine and amide, based on which the authors have proposed a model of the reaction mechanism.

The authors in [17] have described a one-step procedure for the functionalization of graphene oxide with aromatic and non-aromatic amines, such as dibenzylamine, p-phenylenediamine, diisopropylamine and piperidine, using a microwave reaction. The synthesized amine-functionalized materials of graphene oxide (amine-GO) were characterized by spectroscopic techniques including X-ray diffraction (XRD), FTIR, carbon-13 (C13) nuclear magnetic resonance, XPS, transmission electron microscopy (TEM) and thermogravimetric analysis (TGA). Comparative characteristics of analyses have confirmed the functionalization for all amines, achieving relatively high surface atomic nitrogen concentrations of up to 8.8%. The addition of nitrogen-containing fragments will not only change the functionality of GO, but also the chemical, optical and electronic properties, due to the replacement of oxygen-containing groups by nitrogen-containing fragments.

Modified GO particles are widely used in the technology of producing polymer materials for various purposes. The functionalization of GO makes it possible to achieve increased strength, thermal stability, fire safety, etc., due to the chemical interaction of functional groups on the surface of GO with reactive groups of the matrix. In this study [18], a nanocomposite coating based on the epoxy matrix and GO modified with p-phenylenediamine was prepared. The anticorrosion properties of the resulting coating were enhanced by an increase in the degree of filling to 0.1 wt.% due to the chemical interaction between the matrix and amine functional groups; a further decrease in properties is explained by the formation of GO agglomerates. The lowest losses of adhesion were similarly noted for the coating filled with 0.1 wt.% GO, which is explained by the formation of hydrogen bonds between the matrix and the steel substrate. Zhi and colleagues [19] gradually grafted 9,10-Dihydro-9-oxa-10-phosphaphenanthrene-10-oxide and vinyltriethoxysilane (DOPO−VTES−GO) onto the GO surface to improve the thermal stability and fire safety performance of the epoxy matrix. The addition of 5 wt.% DOPO−VTES−GO resulted in an increase in coke yield in the matrix with pristine GO from 19.4 wt.% up to 30.2 wt.% due to the fact that phosphorus accelerates the process of the formation of the coke layer, while silicon improves its stability in the process of the thermal destruction. In a similar way, the functionalization effected the flammability of the polymer by increasing the following indicators: limiting oxygen index (LOI) from 22.3 to 27.5%; the time before the ignition from 88 to 106 s, having reduced the peak heat release rate from 526 to 390 kW/m^2^, as well as the total heat release from 93 to 59 MJ/m^2^. The functionalization of GO changes the structure of the coke layer to a more compact and homogeneous one, which allows us to better protect the epoxy matrix from combustion and prevents the leakage of flammable gases due to the barrier effect. In the study [20], it was possible to increase the main deformation-strength characteristics of the epoxy matrix due to the treatment of GO particles with 3-glycidoxypropyltrimethoxy silane. Tensile, flexural and impact strengths increased by ~10%, ~20% and ~24%, respectively, which is explained by better energy dissipation because of crack deflection when it collides with GO particles.

This paper is devoted to the study of the synthesis conditions and possibilities of the functionalization of electrochemically synthesized graphene oxide with hexamethylenediamine and its use as an effective modifier for epoxy composites. The choice of hexamethylenediamine as a functionalizing agent is due to the fact that it contains functional groups capable of chemical interaction with the functional groups of both GO and the epoxy oligomer [21,22]. The functionalization of nanofillers with various functional reagents containing groups capable of chemical interaction with both polymers and the nanofiller, in most cases, significantly improves the interaction of the nanofiller with the polymer matrix. At the same time, the surface free energy at the nanofiller–polymer interface decreases, while the adhesion increases, which generally results in a significant increase in the deformation-strength properties of polymer composites [23,24,25].

## 2. Materials and Methods

### 2.1. Materials

Dispersed graphite powder (brand code IG-GNP-1) produced in Russia was used in the studies. To obtain polymer composites, we used epoxy diane resin ED-20 and polyethylene polyamine (PEPA) as a hardener, produced by CHIMEX Limited (St. Petersburg, Russia). Tris(1-chloro-2-propyl) phosphate (TCPP) from Xuancheng City Trooyawn Refined Chemical Industry Co., Ltd. (Xuancheng, China) was used to improve the elasticity and reduce the fire hazard of epoxy composites. The presence of phosphorus and chlorine atoms, which are combustion inhibitors in the TCPP molecule, allow us to use it as a fire retardant. The mechanism of reducing the flammability of epoxy composites, when adding TCPP into the composition, was studied by us earlier and described in detail in [26].

### 2.2. Preparation of GO-HMDA

Electrochemical measurements were carried out on a P-150x potentiostat (Elins LLC, Zelenograd, Russia) in a three-electrode cell using a platinum cathode and anode current collector and an 83% H_2_SO_4_ electrolyte. Electrode potentials were measured relative to a mercury sulfate reference electrode (Hg/Hg_2_SO_4_/K_2_SO_4_). Electrochemically oxidized graphite with an amount of electricity of 0.7 A·g·h^−1^ in a galvanostatic mode was washed in double-distilled water (t = 15–18 °C) for 15 min to remove residual sulfuric acid compounds. Electrochemically obtained multilayer graphene oxide (0.1 g) was dispersed in 10 mL of *N*,*N*-dimethylformamide using ultrasonic treatment in a STEGLER 5 DT ultrasonic bath (frequency 40 KHz) at 25 °C for 2 h. The resulting dispersion was refluxed at 90 °C with 0.3 g of 1,3-dicyclohexylcarbodiimide and 1 g of hexamethylenediamine (HMDA) for 10 h. The resulting GO-HMDA was filtered and washed with 100 mL of distilled water and 100 mL of ethanol and dried at 80 °C.

### 2.3. Preparation of Epoxy Nanocomposites

The optimal content of TCPP in the epoxy composition is 40 parts by mass, so the initial epoxy composition had the following composition: ED-20 (100 parts by mass), PEPA (15 parts by mass) and TCPP (40 parts by mass). GO and GO-HMDA were added into the epoxy composition as a nanostructuring agent (0.1 parts by mass). To uniformly distribute and prevent the aggregation of GO and GO-HMDA particles, the epoxy composition was subjected to ultrasonic processing for 30 min at an ultrasonic frequency of 22 ± 2 kHz and an emitter power of 400 W. Before being poured into molds, the epoxy composition was degassed under vacuum for 30 min. Curing of the epoxy composition was carried out in three stages: first, it was cured under natural conditions at room temperature for 24 ± 1 h; after that the samples were successively heat treated for 2 h at 90 ± 5 °C and then 120 ± 5 °C, respectively.

### 2.4. Methods

To record the diffraction patterns of GO and GO-HMDA, the ARL X’TRA instrument (Thermo Scientific, Ecublens, Switzerland) was used with CuKα radiation (λ = 0.15412 nm). Diffraction patterns were recorded in the 2θ range from 5° to 40° at a scanning speed of 2°/min. The surface and structure of the nanostructured graphite particles and the structure of the epoxy composite based on them were studied using a scanning electron microscope with built-in energy dispersive analysis EXplorer (ASPEX, Framingham, MA, USA). Fourier transform infrared spectroscopy (FTIR) was performed on the FT-801 FTIR spectrometer (Simex, Novosibirsk, Russia) in the range of 4000–500 cm^−1^ at room temperature. Raman spectra were recorded using a Renishaw InVia Reflex Raman spectrometer (Wotton-under-Edge, England, UK) equipped with an air-cooled RenCam CCD detector. A laser line with a wavelength of 532 nm was used as an excitation source. To record the spectra, an exposure time of 60 s was used at a power of 1%.

To determine the deformation-strength properties of epoxy composites, a testing machine WDW-5E from Time Group Inc (Beijing, China) was used. The corresponding flexural and tensile strengths were determined according to [27] and [28], respectively, and 5 samples were tested for each group. The impact strength of the epoxy composites was determined according to [29] using an LCT-50D pendulum tester (Beijing United Test Co., Ltd., China) and 5 samples were tested for each group. The heat resistance of the composites according to Vicat softening temperature was determined according to [30] using the B50 method. Kinetic curing curves of epoxy compositions were plotted according to the method described in [31]. Data of differential scanning calorimetry were obtained using a “DTAS-1300” device (Samara, Russia); the mass of the analyzed sample was 20 mg, the heating rate was 16 degrees and the duration was a minute. Thermal resistance of the samples was determined by thermogravimetric analysis using a Q-1500D derivatograph (MOM, Budapest, Hungary) of the Paulik–Paulik–Erdey system; the mass of the analyzed sample was 100 mg, and the studies were carried out in air, in the temperature range of 20–1000 °C and at a heating rate of 10 °C/min.

## 3. Results

**Figure 1:**
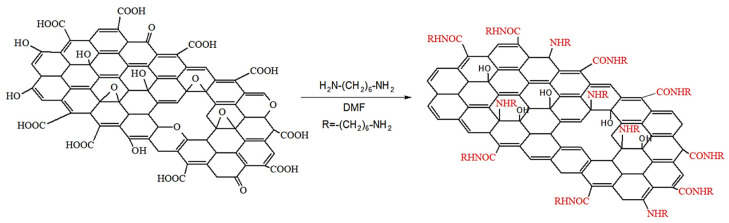
Scheme for obtaining modification of graphene oxide with hexamethylenediamine.

Graphene oxide (multilayer) was obtained by electrochemical (anodic) oxidation of dispersed graphite in sulfuric acid [32]. The surface modification was carried out using the chemical reaction of HMDA and electrochemically synthesized graphene oxide (GO). The probable interaction of graphene oxide with hexamethylenediamine occurs through the mechanism of nucleophilic substitution between the amino group of HMDA and the epoxy groups (-C-O-C-) of GO; the second possible process is the amidation of the carboxyl groups of GO, as shown in Figure 1.

To identify the structure and to study the properties of the synthesized GO and GO-HMDA, methods of IR, IR-Raman spectroscopy and XRD SEM were used.

**Figure 2:**
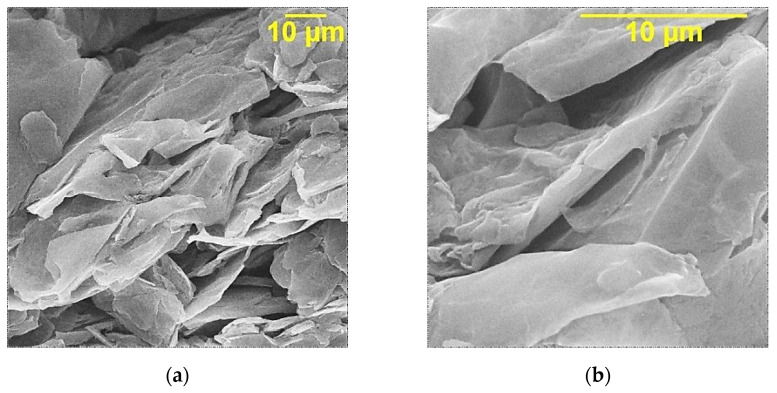

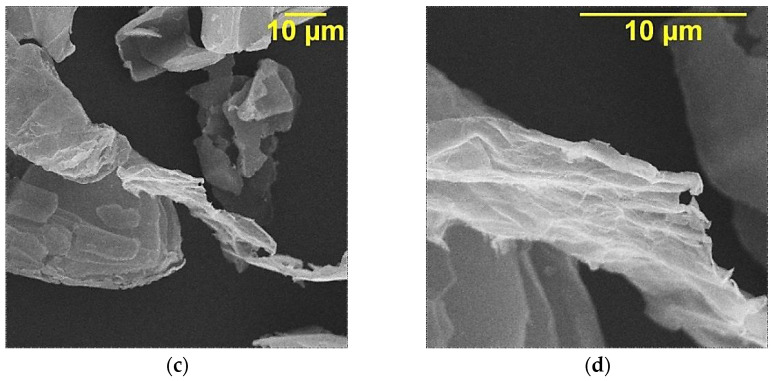
Data of scanning electron microscopy of samples: (**a**)—GO (×5000); (**b**)—GO (×10,000); (**c**)—GO-HMDA (×5000); (**d**)—GO-HMDA (×10,000).

According to the results of scanning electron microscopy, shown in Figure 2c,d, the structure of the GO-HMDA particles is presented by randomly distributed particles, in contrast to the agglomerated clusters of particles of multilayer GO synthesized by the electrochemical method, as shown in Figure 2a,b. It can be assumed that when modified with hexamethylenediamine, partial reduction of multilayer graphene oxide occurs, since the presence of surface oxygen groups contributes to the agglomeration of particles. At the same time, the deformation of the surface of carbon particles increases after the interaction with hexamethylenediamine.

**Figure 3:**
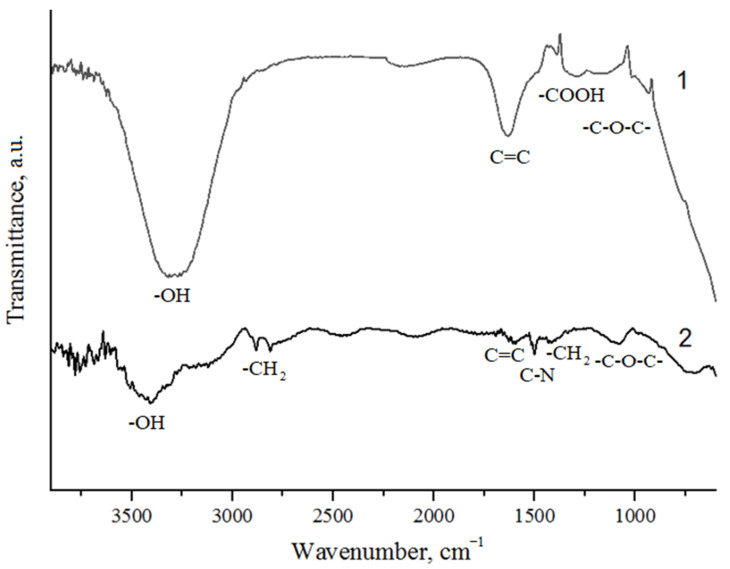
FT-IR spectroscopy: 1—GO; 2—GO-HMDA.

The IR spectrum of GO, shown in Figure 3, shows an intense peak at 3481 cm^−1^, corresponding to vibrations of hydroxyl groups also located between the graphene layers (peak~3300 cm^−1^). There is a peak at 1711 cm^−1^ (C=O group). The peak at 1635 cm^−1^ is due to the presence of sp^2^-hybridization of C=C in the graphene structure. The broad band at ~1103 cm^−1^ corresponds to the bending vibrations of the bonds of epoxy groups [33]. The band at 1350 cm^−1^ represents the bending vibration of -COOH groups. In the IR spectrum of GO-HMDA, the peak corresponding to vibrations of hydroxyl groups shifts to the region of ~3400 cm^−1^, the peak at 1635 cm^−1^ shifts to 1602 cm^−1^, and the peaks at 1711 and 1103 cm^−1^ are not observed, which likely indicates the reduction of carbonyl and epoxy groups. The IR spectrum of GO-HMDA shows peaks at 1534 cm^−1^ and 1248 cm^−1^, which correspond to the bending vibrations of the C-N fragment, and a broad peak of bending vibrations at 1100 cm^−1^ (C-N stretching).

**Figure 4:**
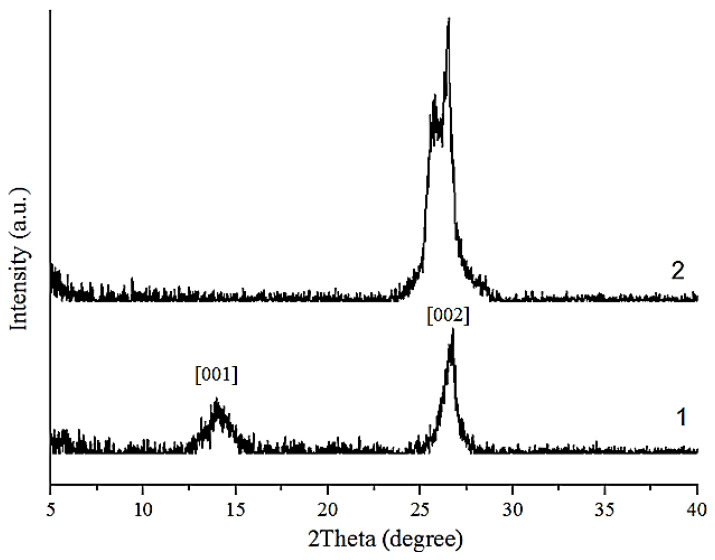
X-ray diffraction: 1—GO; 2—GO-HMDA.

The XRD results in Figure 4 show that a signal with a peak maximum at 2θ = 11.86°, corresponding to the diffraction index of the (001) plane, is recorded in the GO X-ray diffraction pattern. The reflection at 2θ = 26.12° corresponds to the graphite phase (002); however, the peak of the graphene oxide (001) phase is absent in the GO-HMDA diffraction pattern.

**Figure 5:**
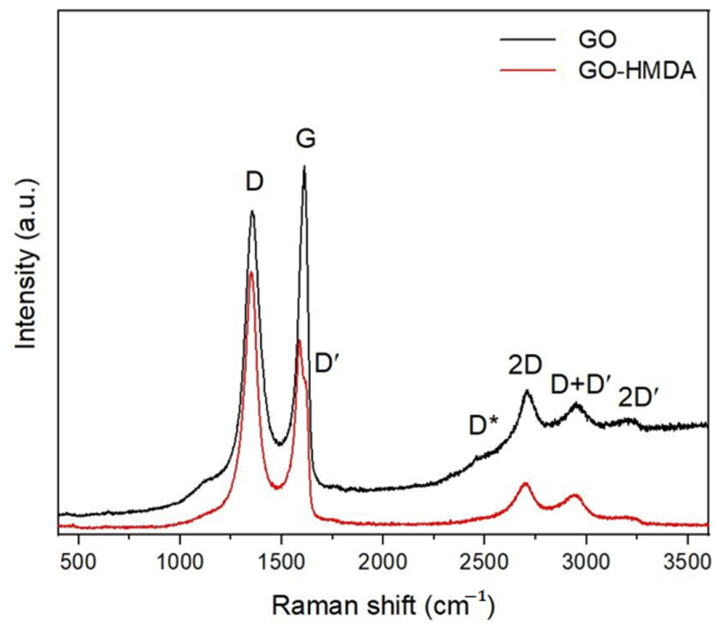
Raman spectra of GO (black) and GO-HMDA (red).

The Raman spectrum of GO is characterized by intense D and G bands and a broad 2D band, as shown in Figure 5, the positions of which are given in Table 1.

The intense G band describes vibrations of the system of sp^2^-hybridized carbon bonds, while the D band indicates the formation of sp^3^-hybridized bonds as a result of graphite oxidation and increased structural defects, as follows from the table. The presence of oxygen atoms causes both an increase in the interplanar distance and a change in the vibration characteristics in the material lattice. For this reason, the Raman spectrum of GO is characterized by more intense D peaks compared to the D peaks of non-oxidized or exfoliated graphites [34].

**Figure 6:**
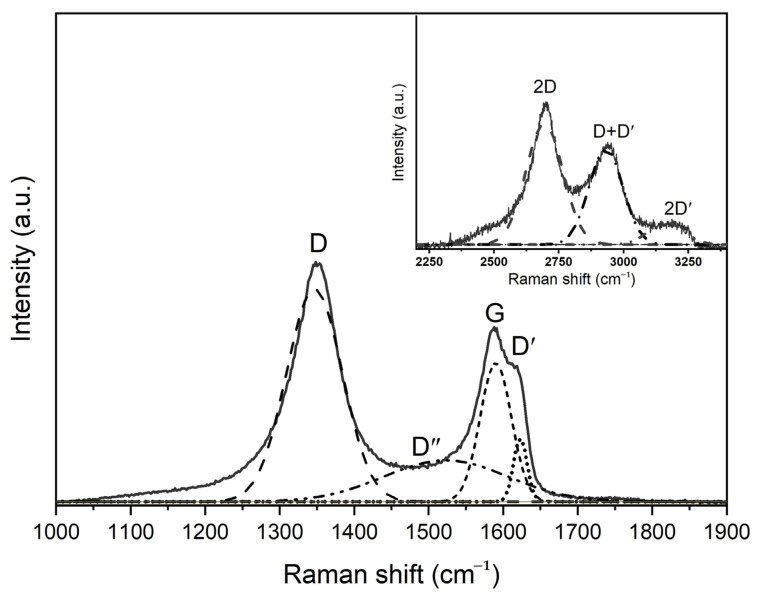
Raman spectrum approximation of GO-HMDA.

The greater the formation of oxygen-containing groups, the greater the intensity of this peak. The spectrum also contains a 2D band, which characterizes the ordered stacking of carbon layers. Depending on the position, shape and intensity in the two-dimensional band obtained as a result of the decomposition of the Raman spectrum into components (Lorentz curves), the number of graphene layers can be determined, as shown in Figure 6.

In the case of a graphene monolayer, the 2D band appears as a single peak near ~2675 cm^−1^. However, with more layers, the 2D band consists of four peaks: D*, 2D, D + D′ and 2D′. In this case, the peak D + D′ is more intense than the others. Increasing the number of layers reduces the intensity of the D* and 2D peaks and shifts the components toward higher wave numbers. The shape of the spectra and the appearance of D*, 2D, D + D′ and 2D′ peaks indicate that GOs are composed of more than two layers of graphene. The ratio of the intensities of the D and G peaks (I_D_/I_G_) can be a measure of randomness in the sp^2^ carbon matrix.

This value can also be used to estimate the size of L_a_ sp^2^ domains in graphene oxide, using the Tuinstra–Koenig relation [35]:

(1) $$L_{a}=(2.4\times 10^{-4})\lambda_{L}^{4}{\left(\frac{I_{D}}{I_{G}}\right)}^{-1}$$

where λ_L_ is the wavelength (nm) of the exciting laser. The values obtained from the quantity formula (for GO, this value is 21.13 nm; for GO-HMDA, it is 14.19 nm) indicate that the sizes of graphene crystallites decrease during the interaction of the functional groups of GO with the amino groups of hexamethylenediamine.

In the Raman spectrum of GO-HMDA, intense D and G bands are also present, but the position of the maxima is shifted toward lower wave numbers, less than those of GO. The G band is accompanied by the D′ shoulder band, indicating a defective structure, which is also confirmed by an increase in the ID/IG ratio from 0.91 in GO to 1.35 in the hexamethylenediamine-modified sample, verifying that the process of GO reduction leads to an increase in structural defects.

The functionalization of nanofillers with various functional reagents containing groups capable of chemical interaction with both polymers and the nanofiller, in most cases, significantly improves the interaction of the nanofiller with the polymer matrix. At the same time, the surface free energy at the nanofiller–polymer interface decreases, while the adhesion increases, which generally results in a significant increase in the deformation-strength properties of polymer composites [23,24,25].

**Figure 7:**
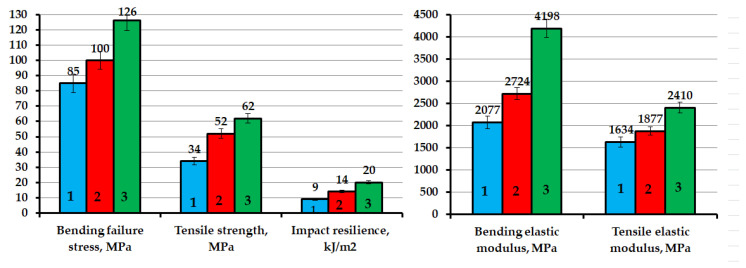
Deformation-strength characteristics of epoxy nanocomposites: 1—100 ED-20 + 40 TCPP + 15 PEPA; 2—100 ED-20 + 40 TCPP + 0.1 GO + 15 PEPA; 3—100 ED-20 + 40 TCPP + 0.1 GO-HMDA + 15PEPA.

The addition of GO-HMDA into the epoxy composition leads to a significant increase in the deformation-strength characteristics of epoxy nanocomposites. At the same time, an increase in the strength index and elastic modulus in bending by 48% and 102%, respectively, was noted. The impact strength index increased by 122%, and an increase in the strength index and tensile elasticity modulus by 82% and 47%, respectively, as compared to the pristine epoxy composite containing no GO-HMDA, was noted, as shown in Figure 7. In addition, the analysis of the obtained results shows that the addition of GO-HMDA has a greater strengthening effect than that of the pristine GO. At the same time, an increase in the deformation-strength properties of the nanocomposites by 19–54% was noted, as compared to the epoxy nanocomposite containing the pristine GO, as shown in Figure 7.

**Figure 8:**
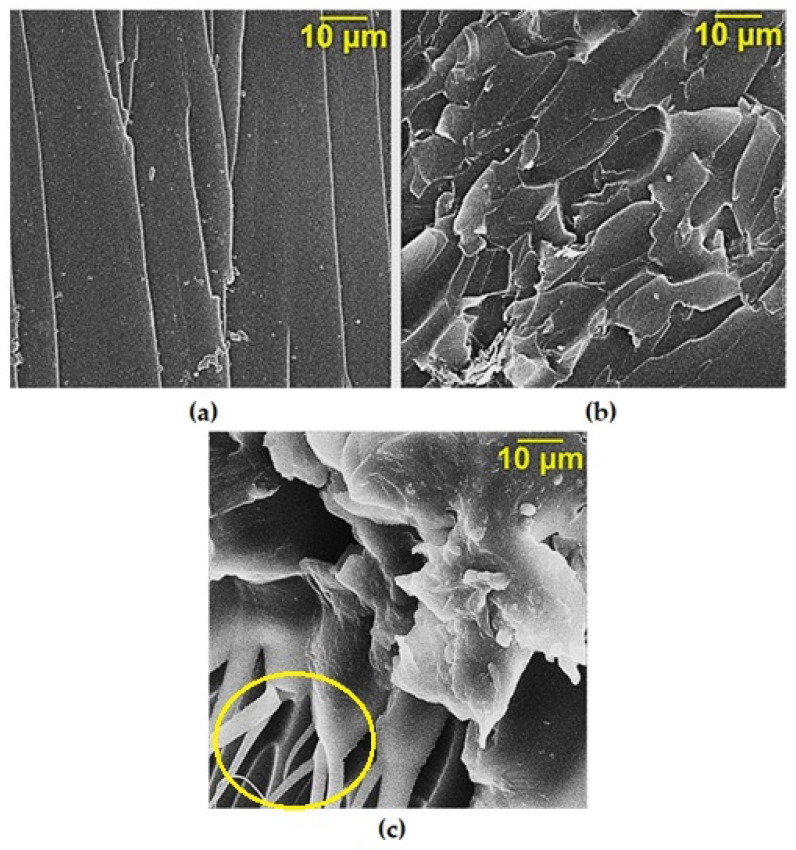
SEM data of the cleavage surface of epoxy nanocomposites. (**a**) Pristine epoxy composite without GO; (**b**) epoxy composite containing GO; (**c**) epoxy composite containing GO-HMDA; the yellow circle—fibrous structures, forming due to the intense stretching of the polymer matrix.

Figure 8a shows the fractography of epoxy resin composite samples without GO which are characterized by a fairly smooth fracture surface, indicating low crack resistance [36,37]. However, when GO is added into the epoxy composition, it affects the morphology of the matrix, increasing the number of defects at the fracture surface, as shown in Figure 8b. This shows an increase in the required energy to destroy the polymer matrix.

The presence of functional groups on the graphene oxide (GO) surface leads to an increase in the depth and number of defects on the cleavage surface, as shown in Figure 8c. This suggests that more energy is required to destroy the system, indicating increased strength and stability [38,39].

Besides a brittle fracture, the epoxy composite also shows local areas indicating the flow of the material during its destruction. This suggests that the material exhibits some level of ductility or plastic deformation before ultimate failure. Furthermore, in certain regions of plastic deformation, pronounced fibrous structures are observed. These fibrous structures are formed due to the intense stretching of the polymer matrix, as shown in Figure 8c.

The presence of increased plastic deformation in the composite material supports the hypothesis that GO–hexamethylenediamine (GO-HMDA) can act as a solid-state hardener. This can be attributed to the functionalization of the GO surface, which results in the formation of a dense cross-linking network around the nanoparticles of GO-HMDA with epoxy groups of the oligomer. This cross-linking network enhances the mechanical properties of the composite, making it more resistant to deformation and failure.

The adsorption interaction of the components of the oligomeric composition with the surface of a solid material (GO) can significantly affect the processes of polymerization and the formation of the phase structure of the material [40,41]. Adsorption can lead to the retention of oligomer molecules on the surface and the slowdown of the polymerization reaction, as well as to changes in the structure of the material, for example, the formation of denser surface layers. Thus, when assessing the effect of a modifying additive on network polymers, it is necessary to take into account adsorption effects, which can change the kinetic and structural properties of the material [42,43,44].

**Figure 9:**
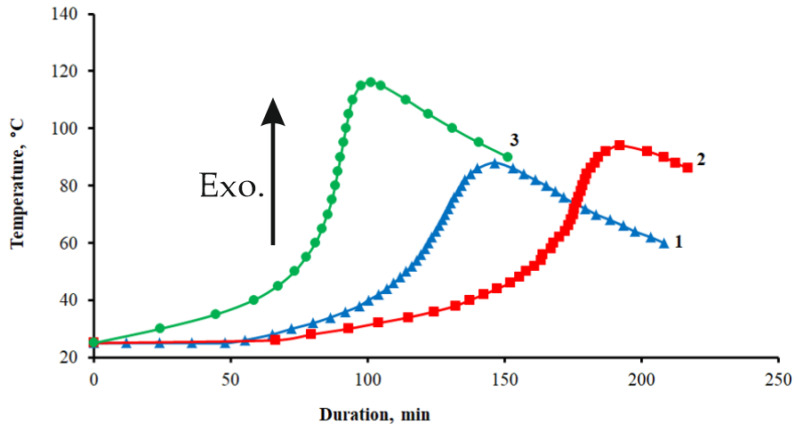
Kinetic curing curves of epoxy compositions: 1—pristine epoxy composition without GO; 2—epoxy composition containing GO; 3—epoxy composition containing GO-HMDA.

The study of the kinetic regularities of curing processes when adding GO and GO-HMDA into the epoxy composition shows that they have different effects on the polymerization processes of the epoxy composite, as shown in Figure 9.

The addition of the pristine GO characterized by high surface activity shows that adsorption results in the retention of epoxy oligomer molecules on the surface of GO, which leads to a slowdown in the rate of the polymerization reaction; this ensures an increase in the duration of the gelation process from 104 to 146 min and of curing from 146 to 195 min, while the maximum self-heating temperature of the sample during curing does not practically change, as shown in Table 2.

The addition of GO-HMDA into the epoxy composition accelerates the curing reaction and increases the amount of heat generated, thus confirming the existence of chemical interaction between the functional groups of HMDA and the epoxy matrix. This fact is proven by a decrease in the duration of the gelation process to 68 min and of curing to 105 min, as well as an increase in the maximum reaction temperature to 116 °C, as shown in Table 2.

**Figure 10:**
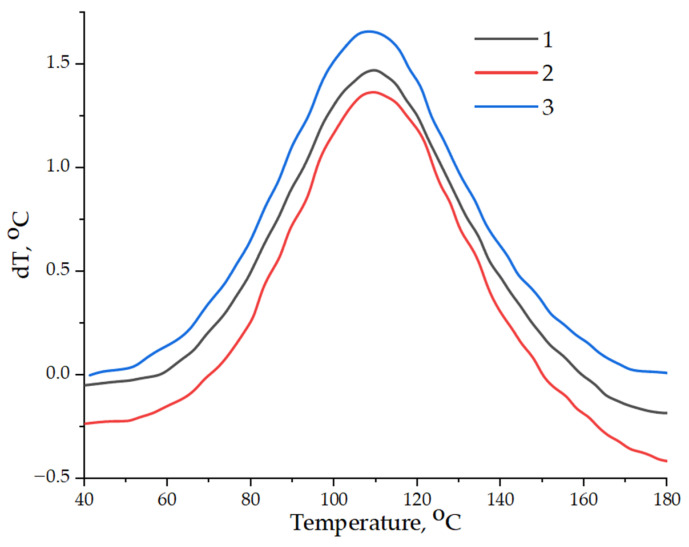
DSC results: 1—pristine epoxy composition without GO; 2—epoxy composition containing GO; 3—epoxy composition containing GO-HMDA.

The data on the curing kinetics obtained using the thermometric method are in good agreement with the data obtained by the DSC method, as shown in Figure 10. The addition of the pristine GO into the epoxy composition slows down the curing process, while the enthalpy of the reaction decreases from 535.7 to 446.4 J/g as a result of increasing the temperature of the onset of curing from 58.9 to 70.7 °C. The addition of GO-HMDA into the epoxy matrix initiates the polymerization process, and the index of reaction enthalpy increases from 535.7 to 614.5 J/g, while the onset temperature decreases from 58.9 to 45.5 °C, as shown in Table 3.

**Figure 11:**
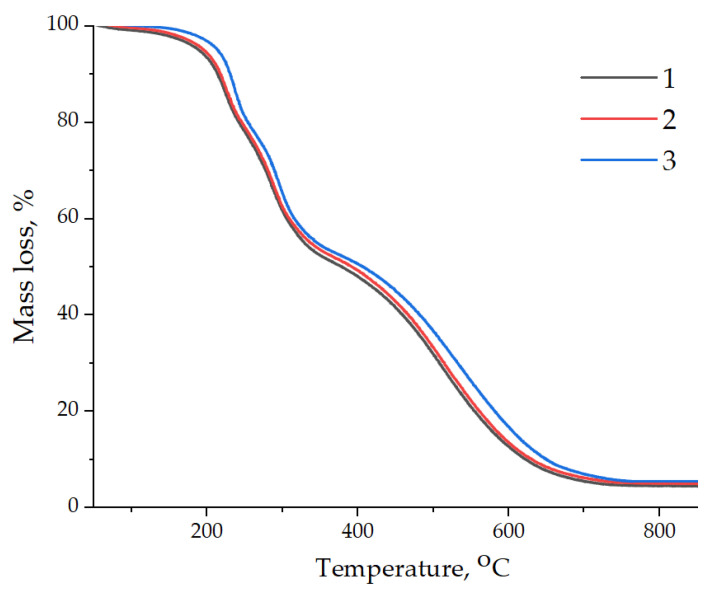
Data of thermogravimetric analysis of samples: 1—pristine epoxy composition without GO; 2—epoxy composition containing GO; 3—epoxy composition containing GO-HMDA.

The effect of adding GO and GO-HMDA into the epoxy composition on the heat resistance and thermal stability of the epoxy composite was studied using thermogravimetry. The obtained thermogravimetric analysis data are presented in Figure 11 and summarized in Table 4.

From the analysis of the data obtained, it follows that the addition of GO does not have a significant effect on the thermal stability of the epoxy composite, while the thermal stability of the composite does not deteriorate throughout the entire temperature range studied (20–900 °C). The addition of GO-HMDA into the epoxy composition provides an increase in the thermal stability of the epoxy composite, while the temperature of the onset of destruction increases from 190–195 to 214 °C. Moreover, an increase in the thermal stability of the epoxy nanocomposites is noted throughout the entire temperature range studied (20–900 °C), which is confirmed by an increase in indicators such as T_10%_–T_80%_. The improvement in the thermal stability of the composites containing GO-HMDA is probably explained by their better dispersion relative to the original GO and the chemical interaction of HMDA with the epoxy matrix, which requires additional energy costs to destroy the cross-linked structure of the epoxy composite [45,46]. It has been found that the addition of both GO and GO-HMDA does not lead to a significant increase in the carbon residue at 900 °C.

## 4. Conclusions

The possibility of chemical redox modification of electrochemically synthesized multilayer graphene oxide with hexamethylenediamine has been investigated. The results of the modification show a reduction in the number of oxygen-containing functional groups on the surface of multilayer graphene oxide and the appearance of amino groups that are capable of chemical interaction with the polymer matrix, which is confirmed by Fourier transform infrared spectroscopy. An increase in the efficiency of carbon particles is also noted. The functionalizing treatment of EGO particles helps reduce the free surface energy at the polymer–nanofiller interface and increase adhesion, which results in improved physical and mechanical characteristics of the composite material. The addition of EGO-HMDA into the epoxy composition leads to a significant increase in the deformation-strength characteristics of the epoxy nanocomposites. At the same time, an increase in the strength index and elastic modulus in bending by 48% and 102%, respectively, is noted. The impact strength index increases by 122%, and an increase in the strength index and tensile elasticity modulus by 82% and 47%, respectively, is noted, compared to the pristine epoxy composite containing no EGO-HMDA. Analysis of the results shows that the addition of EGO-HMDA has a greater strengthening effect than the addition of the pristine GO, while an increase in the deformation-strength properties of the nanocomposites by 19–54% is noted compared to the epoxy nanocomposite containing the pristine GO. The addition of functionalized EGO-HMDA nanoparticles initiates the polymerization process due to the participation of reactive amino groups in the polymerization reaction. This leads to a decrease in the initial curing temperature and an increase in the thermal effect of the polymerization reaction. It has been found that the addition of EGO-HMDA into the epoxy composition contributes to an increase in the thermal stability of the epoxy composite, while the temperature of the onset of destruction increases from 190–195 to 214 °C; an increase in the thermal stability of the epoxy nanocomposites has been found throughout the entire temperature range studied (20–900 °C), which is confirmed by an increase in indicators such as T_10%_–T_80%_.

## Figures and Tables

**Table 1:** Position of D, G and 2D bands in Raman spectra and crystallite size of samples of GO and GO-HMDA.

Sample	Position of the Band (cm^−1^)	Pick Identification	I_D_/I_G_	I_2D_/I_G_	Crystalline Size L_a_ (nm)
GO	1358	D	0.91	0.95	21.13
	1612	G			
	2708	2D			
	2953	D + D′			
	2477	D*			
	3198	2D′			
GO-HMDA	1349	D	1.35	0.29	14.19
	1587	G			
	1616	D′			
	2692	2D			
	2945	D + D′			
	3191	2D′			

**Table 2:** Values of curing indicators of epoxy compositions.

Composition	τ_gel_, min	τ_res_, min	T_max_, °C
Pristine epoxy composition without GO	104	146	88
Epoxy composition containing GO	146	195	94
Epoxy composition containing GO-HMDA	68	105	116

Note: τ_gel_—duration of gelation; τ_res_—duration of curing; T_max_—maximum self-heating temperature of the sample during curing.

**Table 3:** Results of differential scanning calorimetry of epoxy compositions.

Composition	T_start_–T_end_ T_max_ °C	H, J/g
Pristine epoxy composition without GO	58.9–159.5 108.4	535.7
Epoxy composition containing GO	70.7–150.4 107.8	446.4
Epoxy composition containing GO-HMDA	45.5–174.8 108.4	614.5

Note: T_start—_T_end_—the temperature of the onset and the end of the curing process, T_max_—the temperature of the maximum heat release during curing, and H—the thermal effect of reaction.

**Table 4:** The results of the TGA for epoxy nanocomposites.

Samples	T_5%_, °C	T_10%_, °C	T_30%_, °C	T_50%_, °C	T_60%_, °C	T_80%_, °C	Residues at 900 °C, wt.%
EP	190	214	279	385	460	558	4.45
EP/GO	195	217	281	392	467	562	4.85
EP/GO-HMDA	214	231	290	410	486	584	5.51

Note: EP—pristine epoxy composite; EP/GO—epoxy composite containing GO; EP/GO-HMDA—epoxy composite containing GO-HMDA.

## Data Availability

Data are contained within the article.
